# 

**Published:** 1834

**Authors:** 


### CONTENTS OF VOL. VIII.

### No. I.

ART. ESSAYS AND CASES. PAGE.

I. Epidemic Cholera in Indiana. Brief remarks on Epidemic Cholera as it appeared in Versailles, Indiana, and its vicinity, in the summer of 1833, by W. T. S. Cornett, M. D. - - - - - 9

II. Alcohol in Materia Medica. Remarks on the question "Is Alcohol an indispensable article of the Materia Medica," by Edwin A. At Lee, M. D. - - - - - - - - - - - 13

III. Botanical Specimens. Particular Directions for Collecting and Preserving Specimens of Plants, extracted from an unpublished treatise on Practical Botany, by J. L. Riddle, A. M. - - 18

REVIEWS AND BIBLIOGRAPHICAL NOTICES.

IV. Delirium Tremens. Prize Dissertation (for 1831) on Delirium Tremens, by James Conquest Cross, M. D. - - - - - 43

V. Somnambulism. An account of Jane C. Rider, the Springfield Somnambulist, by L. W. Belden, M. D. - - - - - - 62

VI. The Gastric Juice. Experiments and Observations on the Gastric Juice, and the Physiology of Digestion, by William Beaumont, M. D. Surgeon in the U. States Army. - - - 77

VII. Popular Study of Anatomy and Physiology. A projected School and College book of Anatomy and Physiology, designed for both sexes, by the Editor. - - - - - - - - - 99

MISCELLANEOUS INTELLIGENCE.

I. ANALECTIC.

1. Confirmation of Sir Chas. Bell's Opinions on the Functions of the Anterior and Posterior Fasciculi of the Spinal Nerves, 105—2. The Anatomy and Physiology of the Liver, by F. Kierman, M. R. C. S. 108—3. On the efficacy of the Secale Cornutum in Leuchrrhœa, by G. Negri, M. D. 109—4. Herpes, 119—5. Fresh Prepared Carbonate of Iron, ib—6. Ranque's Remedy for swollen Breasts, ib —7. Facial Hemiplegia—External use of Phosphorus, 120—8. Radical cure of Varicocele, 121—9. Case of Hypertrophy of the Muscular Coat of the Stomach, 122—10. Remarks on Local Diseases. Pathology of the Diseases of the Digestive System, by William Stokes, M. D. 123—11. Ointment for cure of Porrigo, 127—12. M. Aubergier's Pomatum for preventing the hair from falling out, ib—13. Improved method of administering Epsom Salts, ib—14. Iodine in Mercurial Salivation, 129—15. Remarks on the Nature of Neuralgias and their Treatment, by M. Piorry, 130—15. On Sanguineous Tumours of the Craneum, 137—16. Stricture of the Rectum treated by the introduction of a Tent, by a new process. By M. Tanchou, 138—17. Advantages of turning the Fœtus by the head rather than by the feet, 141—18. Clinical observations on Fractures of the Neck of the Femur. By Baron Dupuytren, 144—19. On the prevention of Uterine Hæmorrhage, post partem. By Dr. Beatty, 151—20. On the effects of Mammary Irritation in Amenorrhœa. By Dr. Patterson, 153.

II. ORIGINAL.

1. Our Publisher, 155—2. Operative Surgery of the Eye, ib.—3. Cultivation of Medicinal Plants in Ohio, 156—4. Truss for the Permanent Cure of Hernia, ib.—5. Proposed Medical Convention in Columbus, Ohio, 156—6. Catheter and Sound, 158—7. The Locust, 160—8. Summer Residence for the Consumptive, 163—9. Case of Poisoning by Datura Stramonium, 165.

### NO. II.

### ESSAYS AND CASES.

I. Epidemic Cholera and Dysentery at Cincinnati, 1834, by the Editor, 169
II. Dysentery and other Diseases in the Valley of Mill Creek, near Cincinnati, in the summer of 1834, by G. Bailey, M. D. - 194
III. An account of the newly invented Galvanometer, by J. Locke, M. D. 201
IV. A case of partial Amensia, in which the memory for proper names was lost, by the Editor, - - - - - - 209
V. Cases of recovery from wounds of the Brain, by Dr. K. Estep, 212

### REVIEWS AND BIBLIOGRAPHICAL NOTICES.

VI. Illustrations of Pulmonary Consumption, its anatomical character, causes, symptoms and treatment, by G. S. Morton, M. D. - 216
VII. Sylva Americana, or description of American forest trees, by D. J. Browne, - - - - - - - 238
Communication on shade trees, by a Backwoods-man, - - 246

### MISCELLANEOUS INTELLIGENCE.

#### I. Analectic.

1. Sympathy between the Uterus and Mammæ, 249—2. Structure and Functions of the Lymphatic Vessels, 250—3. Pathology of Typhus Fever, 252—4. Follicular Enteritis, 254—5. Chronic Gastritis, 256—6. Treatment of the same, 260—7. Hydrocele cured by Tinctnre of Iodine, 267—8. Resection of the bones in united Fracture, 267—9. Fractured limbs in plaster moulds, 268—10. Bony union in fractures of the neck of the os femoris, 269—11. Lithotomy, 272—12. New Alkaloids, 272—13. Longevity of Medical men, 275—14. Effects of Iodine and Sulphate of Quinine, 277—15. Statistics of Accouchements, 281—16. General statistics of births, ib—17. Treatment of acute rheumatism, 282.

#### II. Analytical.

1. Belladonna in rigidity of the ostincæ, 282—2. Injuries of the head of the fœtus, ib—3. Swollen breasts 283—4. Virtues and proximate elements of ergot, 284—5. Cotton instead of lint for surgical uses, ib—6. Treatment of dry ears with partial deafness, 285—7. Acetate of lead in diseases of the mucous membranes, 287—8. Viola ovata for the bite of the rattle snake, 288—9. Dropsy cured by hydriodate of potash, ib—10. Professional underbidding in England, 289—11. Belladonna in pertussis, 292—12. Ununited fracture treated by friction of the bones, 293—13. Neuralgia cured by galvanism, ib—14. Rice the cause of epidemic Cholera! 295—15. Origin and nature of falling stars, 296—16. Present state of our knowledge of electricity, 298.

#### III. Original.

1. National hospitals in the Valley of the Mississippi, 301—2. Lunatic Asylum of Ohio, 305—3. Library of the Medical College of Ohio, 308—4. Clinical ticket of the Commercial Hospital of Ohio, 310—5. Comparative classes of the Kentucky and Ohio schools, 312—6. Improvements on the Common Trephine. 314—7. Truss for the permanent cure of Hernia, 315—8. Clinical proofs of distinct motory and percepient Nerves, 316—9. Hot and cold baths in alternation, 317—10. Spasmodic action of the Dura Mater, 319—11. Urinary Calculus in a child, 320—12. Meteorological notices, 321—13. Convention of the Physicians of Ohio, 323—14. Epidemic Cholera in Campeache, 324—15. Epidemic Cholera in Butler co. Ohio, 326—16. A Backwoodsman in Philadelphia, 328.

### No. III.

### ESSAYS AND CASES.

I. Western Flora. Synopsis of the Flora of the Western States, by John L. Riddell, - - - - - - - - - 329
II. Northern and Southern Hemispheres. Thoughts on the Moral and Intellectual Energies of Man in the Northern and Southern Hemispheres, by Dr. James Lakey, - - - - - - - - 375
III. Complicated Abdominal Disease. An account of the last illness, death and *post mortem* appearances of the late Dr. Anderson Judkins, of Stubenville, Ohio, by John Andrews, M. D. - - 392
IV. Acetate of Lead in Cholera. Note on the efficacy of the Acetate of Lead in several cases of Epidemic Cholera, in Cincinnati, October, 1834, by the Editor, - - - - - - - - - 402

### MISCELLANEOUS INTELLIGENCE.

I. Analectic.

1. Seat and Nature of Gonorrhœal Ochitis, 409—2. Properties and Effects of the Digitalis Purpurea, ib—3. Treatment of Ileitis, by Wm. Stokes, M. D. 410—4. Treatment of Diarrhœa, by Wm. Stokes, M. D. 417—5. Some Points in the Anatomy, Physiology, and Pathology of the Vertebral Column, 423—6. Experiments on the Sounds of the Heart, 424—7. On the relations of the Cranium to the Organ of Hearing, 425—8. Structure and Functions of the Skin, 426—9. Influence of Gravity and of a Depending Position on the Circulation of the Blood, in Health and in Disease, 427—10. Of the Chemical Properties of the Secretions in Health and Disease, and of the existence of Electrical Currents determined in Organized Bodies by the Acidity and Alkalinity of the Membranes, by M. Donne, 432—11. On Cancer of the Stomach, by Wm. Stokes, M. D. 433—12. On Duodenitis, by Wm. Stokes, M. D. 436—13. On Inflammation of the Jejunum, by Wm. Stokes, M. D. 437—14. On Inflammation of the Illeum, by Wm. Stokes, M. D. 437—15. Inflammation of the Illeum in Children, by Wm. Stokes, M. D. 441—16. On Tabes Mesenterica, by Wm. Stokes, M. D. 443—17. Diseases of the Large Intestines, by Wm. Stokes, M. D. 446—18. On Dysentary, by Wm. Stokes, M. D. 446—19. On the External use of Croton Oil, 447—20. On the Use of Soot in Diseases of the Eyes, 451—21. Successful Treatment of Disunited Fracture by the Tourniquet, 451—22. Luxations of the Humerus, 451—23. Injections of Cold Water into the Umbilical Cord to Promote the Separation of the Placenta, 452—24. Ointment to allay the Irritation of Hæmorrhoidal Tumours, 452.

II. Original.

1. Our Delay, 453—2. Medical Convention of Ohio, 453—3. Gossamer, 482—4. Animal Life in the two Hemispheres, 486—5. Western Flora, 486—6. Whites Improved Stomach Pump, and Cupping Apparatus, 487—7. Small Pox, 487—8. Transylvania University, 488—9. Medical College of Ohio, 488—10. The Backwood's-man in Philadelphia, 488.

---

### No. IV.

### ESSAYS AND CASES.

I. A Synopsis of Western Botany, by J. L. Riddell, - - - 489
II. Remarks on the Utility of Cathartics, by Lewellyin Powell, M. D. 557

### REVIEWS.

III. The American Cyclopædia of Practical Medicine and Surgery, edted by Isaac Hays, M. D. - - - - - - - 575

### MISCELLANEOUS INTELLIGENCE

### I. Analectic.

1. Abstract of Observations on Motions and Sounds of the Heart, by Hugh Carlisle, 585—2. Results of a series of experiments on Revaccination, by Dr. Heim, 590—3. Colchicum in Leucorrhœa, by G. Ritton Esq. 594—4. Treatment of sore nipples, 594—5. Some effects of the Secale Cornutum, 594—6. Sulphuric acid a prophylactic against Saturnine Colic, 595—7. On Creosote, by M. Reichenbach, 595—8. New method of preparing Creosote, by M. Calderini, 598—9. Medicinal properties of Creosote, 598—10. Preservation of Leeches, by M. Bertrand, 599—11. Action of rarified and condensed air on the surface of the body, 600—12. Reunion of the external ear after separation, by D. Mariani, 601—13. Identity of Gonorrhœa and Sipilis, by Hufeland, 602—14. Treatment of Pulmonary Consnmption by Inhalation, by Sendamore, 602—15. Mercurial Inunction in Erysipelas, 609—16. Fatal effusion of blood into the hericardium, by Dr. Carson, 611—17. Ulcers of the Leg, 614—18. Meththods of reducing luxations of the Humerus at the Bristol Infirmary, 615—19. Extraction of the Cataract, by Mr. Guthrie, 616.

### II. Analytical.

1. Observations of change of climate in Consumption by Dr. Huntt,—624 2. Account of Yellow Fever in New Orleans by Dr. Barton, 628—3. Malformation of the Thorax by Dr. Ingles, 634—4. Strichnine locally applied by Prof. Smith, 635—5. Treatment of inverted toe nail by the same, 636—6. Death of Dupuytren, 636

### III. Original.

1. Medical Journals, 637—2. Medical College of Ohio, 638—3. Aphthous Sore Mouth in Adults, 638—4. Cure of Tubercular Consumption and Scrofula 642—5. Willoughly Medical School, 643—6. Medical Graduations, 644—7. Carpenter's Extract of Sarsaparilla 644—8. Galvanic Apparatus for Clinical use 644—9 Cincinnati Medical Society 345—10. Obituary of Dr. Hales, 646.

